# The Chemosensing Role of CatSper in Mammalian Sperm: An Updated Review

**DOI:** 10.3390/cimb45090442

**Published:** 2023-08-23

**Authors:** Sulun Ke, Tao Luo

**Affiliations:** 1 Institute of Life Science, Nanchang University, Nanchang 330031, China; jp4216118047@qmul.ac.uk; 2 Queen Mary School, Medical College, Nanchang University, Nanchang 330031, China; 3 Key Laboratory of Reproductive Physiology and Pathology in Jiangxi Province, Nanchang University, Nanchang 330006, China

**Keywords:** CatSper, chemosensor, endocrine-disrupting chemicals, hyperactivation, progesterone, prostaglandins

## Abstract

After sperm enter the female reproductive tract, the physicochemical and biochemical microenvironment undergoes significant changes. In particular, the large changes in various ions encountered by sperm may alter the physiology of sperm, ultimately compromising capacitation and fertilization. Thus, the rapid response to environmental variations is vital for sperm functions. For example, Calcium, the most crucial ion for sperm functions, enters into sperm via Ca^2+^ permeable ion channels. The cation channel of sperm (CatSper) is a sperm-specific, pH-sensitive, and Ca^2+^-permeable ion channel. It is responsible for the predominant Ca^2+^ entry in mammalian sperm and is involved in nearly every event of sperm to acquire fertilizing capability. In addition, CatSper also serves as a pivotal polymodal chemosensor in mammalian sperm by responding to multiple chemical cues. Physiological chemicals (such as progesterone, prostaglandins, β-defensins, and odorants) provoke Ca^2+^ entry into sperm by activating CatSper and thus triggering sperm functions. Additionally, synthetic and natural chemicals (such as medicines, endocrine disrupting chemicals, drugs of abuse, and antioxidants) affect sperm functions by regulating CatSper-dependent Ca^2+^ signaling. Therefore, understanding the interactions between CatSper and extracellular ligands sheds light on the mechanisms underlying male infertility and offers innovative diagnostic and treatment approaches. This underscores the importance of CatSper as a crucial regulatory target in male reproduction, linking sperm function with the extracellular environment. In conclusion, this review comprehensively summarizes the relevant studies describing the environmental factors that affect CatSper in humans and rodents.

## 1. Introduction

Mammalian sperm gain their fertilizing capacity after undergoing two vital processes, one in the male reproductive tract, known as epididymal maturation, and the other in the female reproductive tract, known as capacitation [1]. After capacitation, sperm hyperactivate with high-amplitude flagellar beats and vigorous movements to swim across the viscous environment in the female reproductive tract [2]. Meanwhile, multiple chemoattractants related to chemotaxis diffusing from the female reproductive tract to navigate sperm toward oocytes [3]. Later on, sperm become competent to undergo the acrosome reaction, which allows sperm to penetrate the zona pellucida (ZP) glycoprotein surrounding the oocytes. Capacitation is contingent upon the influx of Ca^2+^ into the sperm cytoplasm from both intracellular organelles and the extracellular environment [4]. During their journey to oocytes, mammalian sperms encounter a large number of physiological changes, especially the exchanges of ions [5]. Among these ions, Ca^2+^ plays a central role in the regulation of sperm motility, capacitation, hyperactivation, chemotaxis, and acrosome reaction [6]. The changes in sperm function that occur during capacitation depend on a combination of sequential signaling processes where intracellular Ca^2+^ concentration ([Ca^2+^]_i_) plays a central role. From sperm motility to acrosome reaction, [Ca^2+^]_i_ orchestrates these key events of sperm function in the correct time and order [6]. Interestingly, membrane proteins, such as ion channels, ion transporters, and membrane receptors, play a critical role in these events [6].

Specifically, the cation channel of sperm (CatSper) is a sperm-specific, pH-sensitive, and Ca^2+^-permeable ion channel [7]. Crucially, this channel is responsible for the predominant Ca^2+^ entry in mammalian sperm and is involved in nearly every event by which sperm acquire their fertilizing capability. Additionally, ion channels are capable of transporting ions faster than transporters. This allows sperm to respond quickly to guidance cues within the female reproductive tract. Consequently, CatSper enables the translation of large changes in the microenvironment into changes of [Ca^2+^]_i_ [8]. Although fertilization is at the center of creating new life, it is still a long way from being fully understood. A better understanding of the CatSper channel is important, not only to advance our knowledge of the cause of male infertility but also to inspire improvement in the development of male contraceptives. On one hand, the knockout of genes encoding the CatSper channel in male mice, as well as genetic mutations in CatSper genes in humans, lead to male infertility and the inability of sperm to undergo hyperactivation and to penetrate oocytes. On the other hand, CatSper plays a pivotal role in responding to multiple chemical cues, including physiological chemicals (such as progesterone [P4] and prostaglandins [PGs]), and synthetic and natural chemicals (such as medicines and endocrine disrupting chemicals [EDCs]). Therefore, CatSper is also a pivotal polymodal chemosensor in mammalian sperm [9]. Herein, this review comprehensively summarizes the relevant studies describing the physiological, synthetic, and natural chemicals targeting CatSper in humans and rodents.

## 2. Overview of CatSper

CatSper is located in the flagellar principal piece [7]. The CatSper complex consists of four pore-forming alpha subunits (CATSPER1–4) and at least eight auxiliary subunits (CATSPERβ, γ, δ, ε, ζ, and θ [10]; EF-hand calcium binding domain 9; and C2 calcium dependent domain containing 6 [11]) [12,13]. These subunits are conserved between mouse and human, and genetic variations in *CATSPER1*, *CATSPER2*, *CATSPER3*, and *CATSPERE* have been found in infertile men [14,15,16]. These results indicate that CatSper is essential for male fertility in mammals.

Ca^2+^ is crucial in almost every physiological activity by which sperm acquire their fertilizing capability, including motility, capacitation, hyperactivation, the acrosome reaction, and chemotaxis [4]. The knockout of mouse *Catsper* genes results in male infertility and a lack of CatSper current and hyperactivated motility in sperm [17,18,19,20]. In humans, *CATSPER1* and *CATSPER2* mutations have been reported to be involved in asthenoteratozoospermia in men. Their sperm lack the CatSper current, accompanied by lower sperm counts and motility [14,21]. In particular, our recent study found that the copy number variation of *CATSPER2* causes idiopathic male infertility with normal semen parameters [15]. The sperm of this patient had very low CATSPER2 protein expression, no CatSper current, and failed to undergo hyperactivation. In addition, a CATSPER-current-deficient infertile man with a homozygous in-frame deletion in *CATSPERE* showed normal sperm quality but no hyperactivated motility [22,23]. Additionally, *CATSPER3* mutations cause male infertility due to the failure of the acrosome reaction, but there were no defects in routine semen parameters [16]. Therefore, CatSper plays a central role in the fertilizing capacity of sperm.

CatSper cooperates with other sperm ion channels, exchangers, and transporters to regulate sperm functions in mammals. Sperm-specific Na^+^/H^+^ exchangers transport H^+^ out of the sperm while transporting Na^+^ into the sperm plasma membrane, and HV1 expels H^+^ from sperm, creating an alkaline environment within sperm [24]. Correspondingly, the pH-sensitive CatSper is activated, resulting in Ca^2+^ influx and the activation of Ca^2+^-dependent sperm functions. In addition, in mouse sperm, intracellular alkalinization activates KSper, a sperm-specific potassium channel, which further hyperpolarizes the sperm cellular membrane [25]. KSper-dependent membrane hyperpolarization increases the force driving Ca^2+^ influx through CatSper [26]. In humans, KSper-induced hyperpolarization further affects CatSper [26]. Furthermore, Na^+^/K^+^ ATPase α4 functions as transport to maintain the membrane potential and regulate CatSper directly or indirectly [24]. The HCO_3_^−^ transporters (such as SLC26A3) carry HCO_3_^−^ into sperm cells to activate soluble adenylyl cyclase and increase the sperm cyclic adenosine monophosphate (cAMP), which can stimulate CatSper in human and mouse sperm [24].

After sperm enter the female reproductive tract, the physicochemical and biochemical microenvironment undergo significant changes. As a result, the response of sperm to environmental factors is vital for successful fertilization. CatSper is a polymodal chemosensor in mammalian sperm. It plays a pivotal role in responding to multiple chemical cues including P4, PGs, cyclic nucleotides, ZP glycoproteins, serum albumin, β-defensins (DEFBs), and neurotransmitters, drugs, traditional Chinese medicine, EDCs, and antioxidants. Consequently, understanding the diverse mechanisms by which extracellular factors regulate CatSper is of the utmost importance.

## 3. CatSper and Physiological Chemicals

**Figure 1:**
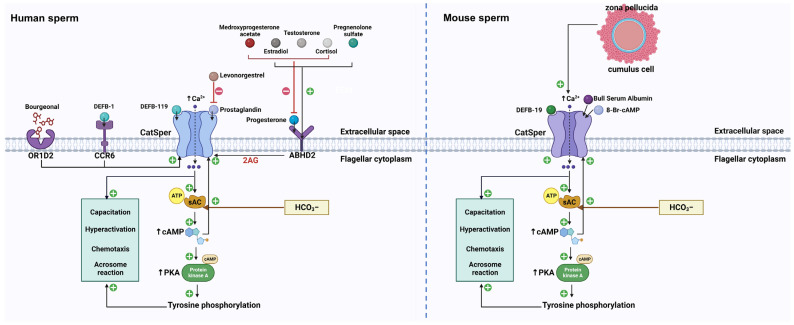
The signaling pathways of different physiological ligands on mammalian CatSper. In human sperm, the P4 binding on the ABDH2 receptor provokes 2-AG depletion and thus removes the inhibition induced by 2-AG on the CatSper channel. Substantially, CatSper opens and allows the extracellular influx of Ca^2+^ and elevates the concentration of intracellular Ca^2+^. The activation of sAC triggered by HCO_3_^−^ and Ca^2+^ increase the level of cAMP, which causes activation of PKA and tyrosine kinase. cAMP can also activate CatSper channel. As a result, the tyrosine phosphorylation leads to sperm capacitation, hyperactivation, chemotaxis, and acrosome reaction. Apart from P4, pregnenolone sulfate can complete the same ABDH2 binding sites to activate CatSper. Cortisol, testosterone, as well as estradiol, target the same binding sites of P4 to activate CatSper. The P4-induced CatSper activation is suppressed by these three molecules, while the PGE-induced CatSper activation is inhibited by levonorgestrel. Also, medroxyprogesterone acetate exerts an inhibitory effect on P4-induced CatSper activation. Additionally, DEFB-1 binding on the CCR6 receptor can activate CatSper and induce Ca^2+^ mobilization. In addition, CatSper is also activated by DEBF-19 and bourgeonal, but OR1D2 is involved in bourgeonal-induced CatSper activation. In mouse sperm, DEFB-19, BSA, as well as 8-BR- cAMP, activate the CatSper channel and induce the mobilization of Ca^2+^ in mouse sperm. Intracellular Ca^2+^ and HCO_3_^−^ can activate sAC and elevate the level of cAMP, leading to the activation of protein kinase A and tyrosine kinases. Thus, the tyrosine phosphorylation initiates a certain process related to sperm function, including sperm capacitation, hyperactivation, chemotaxis, and acrosome reaction. In addition, the interaction between ZP and mouse sperm can elevate intracellular Ca^2+^, which require the CatSper channel to enter mouse sperm. The solid line with the arrow represents activation and the red line represents inhibition. ABDH2: α/β hydrolase domain containing protein 2, 2-AG: 2-arachidonoylglycerol, CCR6: C-C chemokine receptor, DEFB-1: β-defensins 1, DEFB-119: β-defensins 119, DEFB-19: β-defensins 19, OR1D2: olfactory receptor, sAC: soluble adenylyl cyclase, PKA: protein kinase A, BSA: bull serum albumin, ZP: zona pellucida.

In the microenvironment of the female reproductive tract, many physiological chemicals, such as oviducal hormones, regulate sperm functions and increase [Ca^2+^]_i_ [8]. In these hormones, P4 and PGs are the best known for the oviducal ligands of CatSper. They are secreted by the oviduct and serve as the predominant hormones in follicular fluid [8]. In addition, several physiological stimuli, including cyclic nucleotides, ZP glycoproteins, serum albumin, DEFBs, neurotransmitters, and odorant attractants, elicit a CatSper-dependent Ca^2+^ increase [27] (Summarized in Figure 1).

### 3.1. CatSper and Endogenous Steroids

As sperm travel through the female reproductive tract, they are exposed to a variety of steroid hormones. Human follicular fluid (HFF) present in the female reproductive tract is a key factor for human fertilization; it is present at every stage of impregnation. P4, secreted by the oviductal epithelium and cumulus cells, is the predominant hormone in HFF [8]. P4 can elevate [Ca^2+^]_i_ of mammalian sperm [28]. However, the activation of CatSper by P4 has only been reported in humans and rhesus macaques [29]. In murine sperm, P4 cannot activate mouse CatSper, although it increases sperm [Ca^2+^]_i_ [30]. Therefore, CatSper regulation likely occurs via species-specific mechanisms. Under normal physiological conditions, endogenous P4 activates human CatSper through non-genomic actions mediated by the P4/abhydrolase domain containing the 2 (ABHD2)/CatSper/Ca^2+^ axis. This pathway relies on the coordinated action of ABHD2, a P4 receptor expressed in sperm, and endocannabinoid 2-arachidonoylglycerol (2-AG), an endogenous CatSper inhibitor [31]. P4 binds to ABHD2 and triggers the depletion of 2-AG within the sperm plasma membrane and the release of CatSper from 2-AG inhibition, allowing Ca^2+^ influx [31]. P4-induced Ca^2+^ influx triggers multiple Ca^2+^-dependent physiological responses, including hyperactivation, the acrosome reaction, and chemotaxis, which are critical for successful fertilization [30,32].

Another P4-like steroid hormone, pregnenolone sulfate, competes with P4 for the same ADBH2 binding site to activate CatSper in humans [33,34]. In addition, several endogenous steroids, such as testosterone, cortisol, and estradiol, have been identified as CatSper agonists [34]. Interestingly, in human sperm, these steroids target the same P4 binding site to activate CatSper, and they dose-dependently inhibit CatSper-dependent Ca^2+^ influx induced by P4 [35]. A recent study demonstrated that high cortisol levels in human sperm inhibit the P4-induced Ca^2+^ response. The presence of high cortisol levels resulting from anxiety symptoms exerts a competitive inhibitory effect on P4-induced Ca^2+^ influx and the acrosome reaction, ultimately compromising the quality of semen and fertility potential [36]. This suggests that cortisol, a potential stress biomarker, could negatively impact male reproductive health. Interestingly, certain steroids exhibit inhibitory effects on ligand-induced CatSper activation in a selective manner in human sperm [37]. Unlike mibefradil, which inhibits Ca^2+^ influx induced by all steroid hormones, medroxyprogesterone acetate, levonorgestrel, and aldosterone selectively suppress CatSper-dependent Ca^2+^ influx induced by P4, PGs, and the fungal pheromone sirenin in human sperm [37].

### 3.2. CatSper and PGs

Besides P4, PGs are also oviducal ligands for CatSper in human sperm. They are secreted by the oviduct, and the cumulus cells surrounding the oocyte are important ligands for CatSper. In HFF, the coexistence of P4 and PGs could elevate [Ca^2+^]_i_. In addition, seminal fluids contain high concentrations of PGs [38]. In the epididymis, sperm gain their fertilizing ability and maturity, and PGs may affect sperm function during this period. CatSper activation by PGs at the correct time is critical for successful fertilization. During the ejaculatory process, Zn^2+^ in seminal fluid inhibits PG-induced Ca^2+^ influx of human sperm, thereby preventing premature activation of CatSper and facilitating sperm escape into the female genital tract to localize the egg that is ready for fertilization [8]. Prostaglandin E1 (PGE1) activates CatSper, increases [Ca^2+^]_I_ in a biphasic manner with similar amplitudes, and potentiates Ca^2+^ currents similarly to P4 in human sperm [39]. Consistently, PGs do not activate mouse sperm CatSper [30]. This emphasizes the differential regulation of CatSper between humans and mice. Interestingly, there is synergistic activation of human CatSper when PGE-1 and P4 are combined. This result indicates that PGE1 and P4 activate CatSper, apparently through two different binding sites or signaling mechanisms [38,39]. In addition, PGs activate human CatSper with different potencies: PGE1 > PGA1 > PGE2 ≫ PGD2 [30]. However, the mechanism of action of PGs on CatSper has yet to be fully elucidated [9,39].

### 3.3. CatSper and cAMP

The cAMP is a very important physiological chemical and plays a vital role in signaling transduction. In mammalian sperm, the cAMP pathway is essential for sperm functions, such as capacitation and hyperactivation. A study showed that 8-Br-cAMP, an analog of cAMP, increases CatSper-dependent [Ca^2+^]_i_ [40] and modulates P4 to ultimately increase [Ca^2+^]_i_ in mouse sperm [41]. In addition, bicarbonate can activate soluble adenylate cyclase, increase cAMP levels, and stimulate CatSper in human and mouse sperm [42,43,44].

### 3.4. CatSper and ZP Glycoproteins

The ZP acts as a protective matrix surrounding the oocyte in the female reproductive tract. In mammalian fertilization, the interaction between sperm and ZP glycoproteins triggers an increase in sperm [Ca^2+^]_i_ [45]. Xia and Ren [45] found that ZP glycoproteins trigger Ca^2+^ entry into mouse sperm via CatSper. Balbach et al. [46] showed that ZP glycoproteins evoke a rapid increase in intracellular pH, and CatSper translates this change into a Ca^2+^ response in mouse sperm. In addition, sperm from *Catsper1* knockout mice do not exhibit the ZP-glycoprotein-induced [Ca^2+^]_I_ elevation. Indeed, the Ca^2+^ mobilized by ZP glycoproteins requires CatSper to enter sperm, implying that ZP-glycoprotein-induced Ca^2+^ influx is dependent on CatSper [45,47]. The knockout of *Catsper* genes in mice diminishes the ZP penetration and sperm motility. Thus, CatSper is necessary for sperm to penetrate the ZP effectively.

### 3.5. CatSper and Bovine Serum Albumin (BSA)

Capacitation is a functional maturation process that is necessary to produce hyperactivated motile sperm [5]. This process is dependent on extracellular Ca^2+^. BSA can induce sperm capacitation and increase [Ca^2+^]_i_ in several mammals, but these effects are absent in the sperm of *Catsper1*-knockout mice and could be restored by an EGFP-CATSPER1 fusion protein [48]. These results suggest that BSA promotes Ca^2+^ entry into sperm via CatSper.

### 3.6. CatSper and DEFBs

The DEFB family includes small antimicrobial peptides expressed in the reproductive tract and involved in sperm motility and fertilization [49]. DEFB1 was the first identified member of the DEFB family; it is secreted by the epithelium of the male genital tract [50]. In human sperm, DEFB1 binds to its sperm receptor, C-C chemokine receptor 6, and evokes CatSper-dependent Ca^2+^ flux to regulate sperm motility, hyperactivation, and the acrosome reaction [51]. In addition, DEFB19/119 (mouse/human orthologs), secreted by the female germinal duct epithelium and the oocyte-ovarian complex, elicits Ca^2+^ mobilization via CatSper and induces sperm chemotaxis in capacitated sperm [52]. Mouse DEFB19 and human DEFB119 can activate the CatSper current in mouse and human sperm, respectively [52]. *Defb19* knockdown in mouse oviducts and *Defb19* knockout in male mice impairs sperm chemotaxis. In humans, DEFB119 expression and chemotactic activity are markedly decreased in HFF collected from women with idiopathic infertility [52]. These results indicate that DEFB19/DEFB119 play important roles in sperm chemotaxis and are associated with idiopathic infertility.

### 3.7. CatSper and Neurotransmitters

In mammals, receptors for many neurotransmitters and neuromodulators (such as acetylcholine, adenosine, adenosine triphosphate, γ-aminobutyric acid, serotonin, norepinephrine, and dopamine) are found in sperm. Therefore, a sperm is regarded as a neuron with a tail [53,54]. Interestingly, P4 activates CatSper in human sperm via an unconventional endocannabinoid signaling pathway (P4/ABHD2/2-AG/CatSper) [31]. In addition, serotonergic signals enhance hamster sperm hyperactivation via CatSper [55].

### 3.8. CatSper and Odorant Attractants

Sperm chemotaxis guides sperm toward the oocyte and is closely related to sperm capacitation, hyperactivation, the acrosome reaction, and male fertility. In humans, bourgeonal is a typical odorant and chemoattractant that is proposed to activate olfactory receptors (OR1D2) and to open CatSper to increase [Ca^2+^]_i_ via a G-protein-coupled receptor/olfactory G-protein/cAMP/PKA pathway [9,56,57]. Moreover, men with idiopathic infertility and low sensitivity to bourgeonal have decreased OR1D2 protein expression and bourgeonal-activated CatSper current in their sperm [58]. These findings link odor perception to CatSper and male infertility. This sperm odorant attractant may provide a feasible screening method for CatSper-related male infertility.

## 4. CatSper and Medicines

In addition to physiological chemicals, some medicines have been shown to regulate CatSper functions. Some of them negatively affect CatSper, while some traditional Chinese medicines upregulate the expression of CatSper genes and ameliorate sperm function in infertile males (Summarized in Table 1).

### 4.1. CatSper and Traditional Medicine

CatSper is regarded as a primary target for the pharmacological treatment of male infertility and a novel non-hormone target for male contraception. Some traditional medicine has shown promise for improving male infertility through CatSper. Sheng Jing Shan (SJS), a traditional Chinese medicine, has shown efficacy in treating asthenozoospermia. Notably, SJS effectively improved the sperm motility of a rat model of cyclophosphamide (CP)-induced asthenozoospermia by upregulating *Catsper1* expression [59]. Trigonelline semen (TS), also known as fenugreek, is a natural herbal substance recognized for its ability to improve sperm count and motility in infertile men [60]. In a rat model of CP-induced male infertility, TS effectively restored sperm count, motility, testosterone levels, and the expression of *Catsper1*, *Catsper2*, *Catsper3*, and *Catsper4* [61]. *Panax ginseng*, a well-known traditional medicine with multiple pharmacological activities, is beneficial in treating various diseases [62]. Regarding male fertility, studies have noted that mice treated with *P. ginseng* exhibit increased sperm motility and Ca^2+^ levels [63]. *P. ginseng* significantly increases the expression of *Catsper1*, *Catsper2*, *Catsper3*, and *Catsper4* in mouse sperm [63]. A recent investigation reported that a natural herb called *Putranjiva roxburghii* could effectively upregulate the expression of CatSper genes in bull sperm and markedly boost sperm motility [64]. In addition, escanbil is a traditional medicine applied to treat abnormal menstruation and menstrual cramps in folk medicine [65]. It improves sperm motility and alters the expression of CatSper genes in aging mice [66]. These results suggest that CatSper may be a potential therapeutic agent for natural medicine treatment of male infertility.

CatSper has attracted worldwide attention as a novel non-hormone target for male contraception. Matrine, a traditional Chinese medicine used for cancer treatment, has multiple effects, such as antiviral, antitumor, and insecticidal activities. We showed that matrine downregulated the expression of *Catsper1*, *Catsper2*, *Catsper3*, and *Catsper4* in mouse testes. It decreased sperm CatSper current to disrupt a series of Ca^2+^-dependent sperm activities including motility, capacitation, and the P4-induced acrosome reaction [67]. We also found that another traditional medicine, anethole, suppressed CatSper current and inhibited the ability of human sperm to respond to P4 [68]. *Rhynchosia volubilis*, a traditional Chinese herb, is a major component of folk contraceptive prescriptions in China. Interestingly, we identified a new compound (rhynchone A) from *R. volubilis*. Rhynchone A could activate CatSper and induce Ca^2+^ signaling but suppress P4-induced Ca^2+^ signaling in human sperm [69]. In addition, two plant triterpenes, pristimerin and lupeol, were reported to inhibit P4 activation of human CatSper and are potential candidates for non-hormonal male contraception [33]. However, subsequent studies have contradicted these findings and confirmed that pristimerin and lupeol cannot inhibit CatSper activation in human sperm [34,35,70].

### 4.2. CatSper and Anti-Depressants

Selective serotonin reuptake inhibitors are the most widely used antidepressants in the United States and Europe, but recent research has highlighted their potential to impair male fertility [71]. Researchers have demonstrated that sertraline inhibits CatSper currents induced by intracellular alkalinization, voltage changes, P4, and PGs in human sperm [72]. Sertraline has a significant inhibitory effect on the acrosome reaction and viscous-medium penetration induced by P4 and PGs [72]. These findings suggest that the therapeutic administration of sertraline for depression may impair human reproduction.

### 4.3. CatSper and 5-Alpha Reductase Inhibitors

Finasteride (FS) and dutasteride (DS), two 5-alpha reductase inhibitors, are widely used to treat benign prostate hyperplasia. However, their prolonged use has been shown to adversely affect male semen quality [73]. FS activates CatSper, at least partially, via PG binding sites, whereas DS activates CatSper, at least partially, through P4 binding sites in human sperm [74]. Thus, they interfere with Ca^2+^ signaling mediated by PGs and P4. In addition, the exposure of mice to DS affected sperm count and motility and the expression of CatSper genes in caput and caudal epididymal sperm [75].

### 4.4. CatSper and Analgesics

Paracetamol is widely used as a mild analgesic to alleviate fever and pain. However, rodent studies have shown that paracetamol may have negative effects on sperm count and motility due to its endocrine effects. Additionally, high concentrations of paracetamol in male urine have been linked to lower sperm motility [76]. In human sperm, paracetamol is metabolized to N-arachidonoylphenolamine via fatty acid amide hydrolase expressed in the sperm neck region. N-arachidonoylphenolamine directly activates human CatSper, reduces sperm motility, and affects viscous-medium penetration [77].

### 4.5. CatSper and Ca^2+^ Channel Blockers

Nifedipine is a Ca^2+^ channel blocker and is used as an antihypertensive medicine. It exhibits anti-fertility effects in male rats, resulting in a significant reduction in sperm motility and count [78]. Nifedipine treatment reduces sperm motility and count and substantially downregulates the expression of CatSper genes in mouse epididymal sperm [75]. In addition, RU1968, a steroid-based selective and potent cross-species inhibitor of CatSper, has been demonstrated to suppress the activation of CatSper in human, mouse, and sea urchin sperm [79]. Therefore, nifedipine serves as a powerful tool for the investigation of the physiological function of CatSper in human sperm and for the promotion of the development of non-hormonal male contraceptives.

### 4.6. CatSper and Phosphodiesterase (PDE)-Inhibitors

Trequinsin hydrochloride, a PDE-3 inhibitor, has emerged as a promising CatSper agonist. In human sperm, trequinsin hydrochloride exhibits a P4-like agonist profile and significantly potentiates the CatSper current, effectively increasing sperm hyperactivation and viscous-medium penetration [80]. Additionally, this CatSper agonist induces a concentration-dependent elevation in Ca^2+^ levels through cross-desensitization with PGE1 [80].

### 4.7. CatSper and Anti-Inflammatory Drugs

Cisplatin is the most widely used drug in oncology treatment. However, cisplatin-based treatment of testicular cancer disrupts spermatogenesis and reduces the sperm motility of patients [81]. The indole derivative N′-(4-dimethylaminobenzylidene)-2-1-(4-(methylsulfinyl) benzylidene)-5-fluoro-2-methyl-1H-inden-3-yl) acetohydrazide (MMINA) has significant anti-inflammatory and antioxidant effects and can protect against the testicular toxicity induced by cisplatin [82]. Most importantly, MMINA activates CatSper by upregulating the expression of CatSper genes in rat sperm [83]. Moreover, MMINA is capable of forming hydrogen bonds with CatSper [83].

## 5. CatSper and EDCs

CatSper is not only a chemosensor for physiological chemicals and medicines, but is also a target for environmental chemicals. EDCs, a group of chemicals found in the environment, food, and consumer products, interfere with human hormone synthesis, metabolism, and reproduction. CatSper harbors binding sites for structurally diverse EDCs that potentially impact natural fertilization in several ways [84] (Summarized in Table 2).

### 5.1. CatSper and Environmental Estrogens

Initially, EDCs were called xenoestrogens due to their estrogenic, antiestrogenic, androgenic, and antiandrogenic effects [85]. Steviol, a natural non-caloric sweetener metabolite, exerts endocrine effects on human sperm by antagonizing P4 and agonizing CatSper, resulting in a rapid influx of Ca^2+^ [86]. Bisphenol A (BPA), a ubiquitous EDC and synthetic organic compound, has been significantly and negatively associated with male fertility [87]. BPA binds to estrogen receptors α and β and exhibits estrogenic activity [88]. Animal studies have revealed that BPA impairs sperm function by reducing the expression of CatSper genes and the CatSper current [89]. In GC-2 cells, a mouse spermatogonia cell line, BPA decreased the growth rate and [Ca^2+^]_i_, and downregulated the expression of *Catsper1*, *Catsper2*, *Catsper3*, and *Catsper4* through Ten-eleven translocation 1 [90]. In humans, bisphenol A diglycidyl ether and bisphenol analogs—but not BPA—activate CatSper [91]. Our study showed that diethylstilbestrol, a well-known, synthetic, non-steroidal estrogen, potentiates CatSper currents, increases the [Ca^2+^]_i_, and inhibits P4-induced Ca^2+^ influx and sperm functions in humans [92]. Perfluorooctane acid, an organic pollutant, activates CatSper to elevate the [Ca^2+^]_i_ in human sperm [93]. Like diethylstilbestrol, perfluorooctane acid suppresses the P4-induced CatSper current, Ca^2+^ influx, and sperm functions [93]. In addition, the diversity of EDCs implies that even heavy metals may possess estrogenic activity. Cadmium is considered an EDC with significant toxicity to the reproductive system; it acts as an estrogen mimic and has the ability to bind ERs [94]. Cadmium impairs sperm function via a CatSper-mediated mechanism by affecting the expression of CatSper genes in mice [95].

### 5.2. CatSper and Pesticides

p,p′-Dichlorodiphenyldichloroethylene, a metabolite of dichloro-diphenyl-trichloroethane commonly found in human reproductive fluids, activates CatSper to induce Ca^2+^ entry into sperm and disrupts acrosome reaction [96]. Pentachlorophenol, a widely used pesticide, suppresses the P4-induced CatSper current, Ca^2+^ influx, and sperm functions in humans [97]. Recently, a study investigated the effect of 53 pesticides and pesticide metabolites on human sperm. The results demonstrated that, although 26 pesticides activated CatSper and interfered with signaling triggered by P4 and PGs, they may interact with the unique binding sites or the P4 and PG binding sites of CatSper [98]. Thus, pesticides, either alone or in low-dose mixtures, have the potential to negatively affect sperm function by interfering with normal Ca^2+^ signaling in human sperm via CatSper.

### 5.3. CatSper and Chemical Ultraviolet (UV) Filters

Chemical UV filters, commonly present in daily-use sunscreens, are among the most potent triggers of Ca^2+^ signaling. They directly activate CatSper in human sperm and elevate [Ca^2+^]_i_ [84]. A recent study investigated the effect of 31 chemical UV filters approved in the European Union and the United States on human sperm. Although 29 of the 31 chemical UV filters induced Ca^2+^ signaling in human sperm, only nine of these chemicals could activate CatSper, including 4-Methylbenzylidene camphor, 3-Benzylidene camphor, meradimate, amiloxate, octisalate, benzylidene camphor sulfonic acid, homosalate, benzophenone-3, and octinoxate [99]. Of these chemicals, 3-Benzylidene camphor, benzylidene camphor sulfonic acid, and 4-Methylbenzylidene camphor have been found to competitively inhibit P4-induced Ca^2+^ signaling and target its binding sites in CatSper [84,99]. These results suggest that some chemical UV filters have the potential to interfere with P4-induced Ca^2+^ signaling and negatively affect sperm functions.

## 6. CatSper and Drugs of Abuse

Interestingly, some addictive drugs affect sperm functions through CatSper. Methamphetamine (METH) is a highly addictive central nervous system stimulant that has detrimental effects on male reproductive health, including impaired spermatogenesis, testicular damage, and abnormal sperm quality [100]. In particular, a novel investigation showed that rats receiving METH resulted in a decrease in testis and epididymis weight [101]. Meanwhile, the relative expression levels of *Catsper1*, *Catsper2*, *Catsper3*, and *Catsper4*, as well as the sperm motility associated gene *Mvh*, were decreased significantly [101]. In addition, the exclusive expression of *Catsper1–4* in testes is required for sperm motility and fertility [21,102]. As a result, the downregulation of these genes induced by METH increases the possibility of male infertility. Therefore, men addicted to METH may encounter potential reproductive problems.

Ketamine, a dissociative anesthetic widely used in human and animal medicine, has become a popular recreational drug because it can induce hallucinatory effects. We showed that ketamine affects sperm motility, viscous-medium penetration, and the P4-induced acrosome reaction by inhibiting CatSper in human sperm, thus decreasing [Ca^2+^]_i_ [103]. In addition, ketamine is an antagonist of the N-Methyl-D-aspartic acid (NMDA) receptor. In our recent study, we found that the NMDA receptor is expressed in human sperm and involved in the inhibitory effect of ketamine on human sperm functions [104]. Specifically, NMDA, the physiological ligand of NMDA, could partly alleviate the motility of human sperm and significantly recover the capacitation and acrosome reaction, as well as [Ca^2+^]_i_ [104]. Therefore, the competitive receptor binding between ketamine and NMDA may provide novel insight for clinical diagnoses of ketamine abusers. Collectively, CatSper-related drugs of abuse have been implicated in impaired sperm function and/or male infertility.

## 7. CatSper and Antioxidants

Oxidative stress occurs when the generation of reactive oxygen species (ROS) exceeds the natural antioxidant defenses of bodies. Thus, the precise balance of ROS and antioxidants within sperm are necessary for capacitation and fertilization. The major effect of oxidative stress compromising sperm function is caused by two principal mechanisms, DNA damage and lipid peroxidation [105]. In human sperm, ROS damages DNA directly by the production of 1,N^6^-ethenoadenosine and 1,N^2^-ethenoguanosine, resulting in DNA structure instability and leading to single-strand breaks [106]. Once the transcription and translation of post-spermiogenesis stop, the DNA repair during developing sperm is terminated [107]. Hence, sperm function and pregnancy outcome are strongly impacted.

To counteract ROS damage, the human body has developed a variety of antioxidant strategies. For instance, non-enzymatic antioxidants contained within the seminal fluid, like vitamin E and selenium [108]. Interestingly, treatment with these two antioxidants upregulates the expression of *Catsper* in the testes of young adult and aged male mice, which are the genes responsible for sperm motility [109,110]. Meanwhile, sperm parameters such as viability rate and morphology also show an improvement after treatment [109,110]. Consequently, these two essential components play a crucial role in the maintenance of male reproduction.

## 8. Conclusions

CatSper plays a key role in male fertility by controlling extracellular Ca^2+^ influx into sperm and it is also a polymodal chemosensor that harbors structurally diverse binding sites for extracellular factors. CatSper regulates key sperm physiological processes, including capacitation, hyperactivation, the acrosome reaction, and chemotaxis, by responding to physiological ligands. These processes are necessary to produce sperm capable of fertilization. In addition, the interaction between CatSper and extracellular factors, such as EDCs and drugs, may potentially disrupt sperm function and induce male infertility [111]. Consequently, CatSper is a crucial regulatory target for male fertility. It links sperm function with the external environment. Therefore, CatSper is regarded as a primary target for the pharmacological treatment of male infertility. Future investigation is warranted to develop highly specific and effective CatSper inhibitors, which could serve as an alternative approach to male hormonal contraception. Although we know that all these mentioned ligands have different effects on the opening of CatSper, many questions about the physiology of CatSper remain unanswered; in particular, the mechanism by which these ligands directly or indirectly modulate CatSper is unclear. In addition, there is still a lack of understanding of how the interactions that exist between ligands affect CatSper regulation. Therefore, it is necessary to understand CatSper in a more physiological manner. Further studies should be based on the molecular basis of ligands during binding CatSper. This could facilitate the elucidation of how the CatSper-based pathway regulates multiple ligands within mammalian sperm.

## Figures and Tables

**Table 1:** The effect of different medicines on CatSper channel and sperm function.

No.	Medicines	Classification	Species	Effects on CatSper	Effects on Sperm Function
1	Sheng-Jing-Shan	Traditional medicine	Mouse	*Catsper1* expression ↑	Motility ↑
2	Trigonellae Semen	Traditional medicine	Mouse	*Catsper1–4* expression ↑	Motility ↑, Count ↑, Testosterone ↑
3	Panax ginseng	Traditional medicine	Mouse	*Catsper1–4* expression ↑	Motility ↑
4	Putranjiva roxburghii	Natural herb	Bull	CatSper gene expression ↑	Motility ↑
5	Escanbil	Traditional medicine	Mouse	CatSper gene expression ↑	Motility ↑
6	Matrine	Traditional medicine	Mouse	CatSper currents ↓	Motility ↓, Capacitation ↓, P4-induced AR ↓
				*Catsper1–4* expression ↓	
7	Anethole	Traditional medicine	Human	CatSper currents ↓	P4-induced AR ↓
8	Rhynchosia volubilis	Traditional medicine	Human	CatSper currents ↑	P4-induced Ca^2+^ influx ↓
9	Sertraline	Antidepressants	Human	CatSper currents ↓	P4, PGEs-induced AR & Penetration ↓
10	Finasteride	5-α reductase inhibitor	Human	CatSper activation	PGE-induced Ca^2+^ influx ↓
11	Dutasteride	5-α reductase inhibitor	Human	Human: CatSper current	Human: P4-induced Ca^2+^ influx ↓
			Mouse	Mouse: CatSper gene expression ↓	Mouse: Motility ↓, Count ↓
12	N-arachidonoyl phenolamine	Analgesic	Human	CatSper activation	Motility ↓, Penetration ↓
13	Nifedipine	Ca^2+^ channel blockers	Mouse	CatSper gene expression ↓	Motility ↓ Count ↓
14	RU1968	Ca^2+^ channel blockers	Human, Mouse	CatSper currents ↓	Motility ↓, Hyperactivation ↓
15	Trequinsin hydrochloride	PDE-inhibitor	Human	CatSper currents ↑	Hyperactivation ↑, Penentration ↑
16	MMINA	Anti-inflammatory medicine	Mouse	CatSper currents ↑	Motility ↑, Count ↑
				CatSper gene expression ↑	
17	Methylamphetamine	Central nervous system stimulant	Mouse	*Catsper1–4* expression ↓	Motility ↓, Fertility ↓
18	Ketamine	Anesthetic	Human	CatSper currents ↓	Motility ↓, Penetration ↓, P4-induced AR ↓

P4: progesterone, AR: acrosome reaction, PGE: prostaglandin E, “↑” indicates increase, “↓” indicates decrease.

**Table 2:** The effect of different EDCs on CatSper and sperm function.

NO.	EDCs	Species	Effects on CatSper	Effects on Sperm Function
1	Steviol	Human	CatSper activation	Antagonize P4
2	Bisphenol A	Mouse	CatSper currents ↓	Motility ↓, Spontaneous AR ↓, P4-induced AR ↓
			*Catsper1–4* expression ↓	
3	BADE	Human	CatSper activation	P4-induced Ca^2+^ influx ↓
4	Diethylstilbestrol	Human	CatSper currents ↑	P4-induced Ca^2+^ influx, AR and Penetration ↓
5	PFOA	Human	CatSper currents ↑	P4-induced Ca^2+^ influx, AR and Penetration ↓
6	Cadmium	Mouse	CatSper currents ↓	Motility ↓, P4-induced and Spontaneous AR ↓
			CatSper gene expression ↓	
8	p,p′-DDE	Human	CatSper currents ↑	Spontaneous AR ↓
9	Pentachlorophenol	Human	CatSper activation	Spontaneous AR ↓
				P4 and PGE-induced Ca^2+^ influx ↓
				P4-induced hyperactivation and Penetration ↓
10	Milbemectin A4	Human	CatSper activation	P4 and PGE-induced Ca^2+^ influx ↓
11	Milbemectin A3	Human	CatSper activation	P4 and PGE-induced Ca^2+^ influx ↓
12	Chlorpyrifos	Human	CatSper activation	P4 and PGE-induced Ca^2+^ influx ↓
13	Prosulfocarb	Human	CatSper activation	P4 and PGE-induced Ca^2+^ influx ↓
14	Fipronil Sulfone	Human	CatSper activation	P4 and PGE-induced Ca^2+^ influx ↓
15	Trifluralin	Human	CatSper activation	P4 and PGE-induced Ca^2+^ influx ↓
16	Endosulfan	Human	CatSper activation	P4 and PGE-induced Ca^2+^ influx ↓
17	Metofluthrin	Human	CatSper activation	P4 and PGE-induced Ca^2+^ influx ↓
18	Imazalil	Human	CatSper activation	P4 and PGE-induced Ca^2+^ influx ↓
19	Pyraclostrobin	Human	CatSper activation	P4 and PGE-induced Ca^2+^ influx ↓
20	Fenitrothion	Human	CatSper activation	P4 and PGE-induced Ca^2+^ influx ↓
21	Oxadiazon	Human	CatSper activation	P4 and PGE-induced Ca^2+^ influx ↓
22	Lindane	Human	CatSper activation	P4 and PGE-induced Ca^2+^ influx ↓
23	Prochloraz	Human	CatSper activation	P4 and PGE-induced Ca^2+^ influx ↓
24	Cypermethrin	Human	CatSper activation	P4 and PGE-induced Ca^2+^ influx ↓
25	Propiconazole	Human	CatSper activation	P4 and PGE-induced Ca^2+^ influx ↓
26	Chlorothalonil	Human	CatSper activation	P4 and PGE-induced Ca^2+^ influx ↓
27	Permethrin	Human	CatSper activation	P4 and PGE-induced Ca^2+^ influx ↓
28	Tebuconazole	Human	CatSper activation	P4 and PGE-induced Ca^2+^ influx ↓
29	dPTA	Human	CatSper activation	P4 and PGE-induced Ca^2+^ influx ↓
30	Boscalid	Human	CatSper activation	P4 and PGE-induced Ca^2+^ influx ↓
31	Triticonazole	Human	CatSper activation	P4 and PGE-induced Ca^2+^ influx ↓
32	PBS	Human	CatSper activation	P4 and PGE-induced Ca^2+^ influx ↓
33	Cyprodinil	Human	CatSper activation	P4 and PGE-induced Ca^2+^ influx ↓
34	Prothioconazole	Human	CatSper activation	P4 and PGE-induced Ca^2+^ influx ↓
35	4-MBC	Human	CatSper activation	P4-induced Ca^2+^ influx ↓
36	3-BC	Human	CatSper activation	P4-induced Ca^2+^ influx ↓
37	Meradimate	Human	CatSper activation	P4-induced Ca^2+^ influx ↓
38	Amiloxate	Human	CatSper activation	P4-induced Ca^2+^ influx ↓
39	Octisalate	Human	CatSper activation	P4-induced Ca^2+^ influx ↓
40	BCSA	Human	CatSper activation	P4-induced Ca^2+^ influx ↓
41	Homosalate	Human	CatSper activation	P4-induced Ca^2+^ influx ↓
42	Benzophenone-3	Human	CatSper activation	P4-induced Ca^2+^ influx ↓
43	Octinoxate	Human	CatSper activation	P4-induced Ca^2+^ influx ↓

3-BC, 3-Benzylidene camphor; 4-MBC, 4-Methylbenzylidene camphor; BADE, Bisphenol A diglycidyl ether; BCSA, Benzylidene camphor sulfonic acid; dPTA, Desthioprothioconazole; PBA, 3-Phenoxybenzoic acid; PFOA, Perflfluorooctane acid; p,p′-DDE, p,p′-Dichlorodiphenyldichloroethylene, “↑” indicates increase, “↓” indicates decrease.

## Data Availability

Not applicable.
